# Glandular papilloma in lung parenchyma

**DOI:** 10.1002/rcr2.1389

**Published:** 2024-05-21

**Authors:** Minlong Zhang, Yinghua Guo, Cuiping Yang

**Affiliations:** ^1^ College of Pulmonary & Critical Care Medicine, 8th Medical Centre Chinese PLA General Hospital Beijing People's Republic of China

**Keywords:** computed tomography, glandular papilloma, lung tissue

## Abstract

Solitary respiratory papilloma is a rare epithelial tumour that can be categorized into multiple subtypes. The glandular type (Glandular papilloma, GP) is the rarest. Most GP occurs in the proximal airways and is only rarely found in the lung parenchyma. In this article, we reported a case of GP in lung parenchyma.

## Graphical abstract

We report a rare case of glandular papilloma in lung parenchyma. The glandular type of solitary respiratory papilloma (Glandular papilloma, GP) is the rarest type of these rare epithelial tumours. Most GP occurs in the proximal airways and is only rarely found in the lung parenchyma.

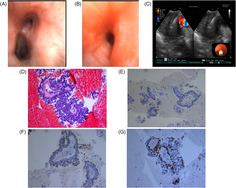

## CLINICAL IMAGE

**FIGURE 1:**
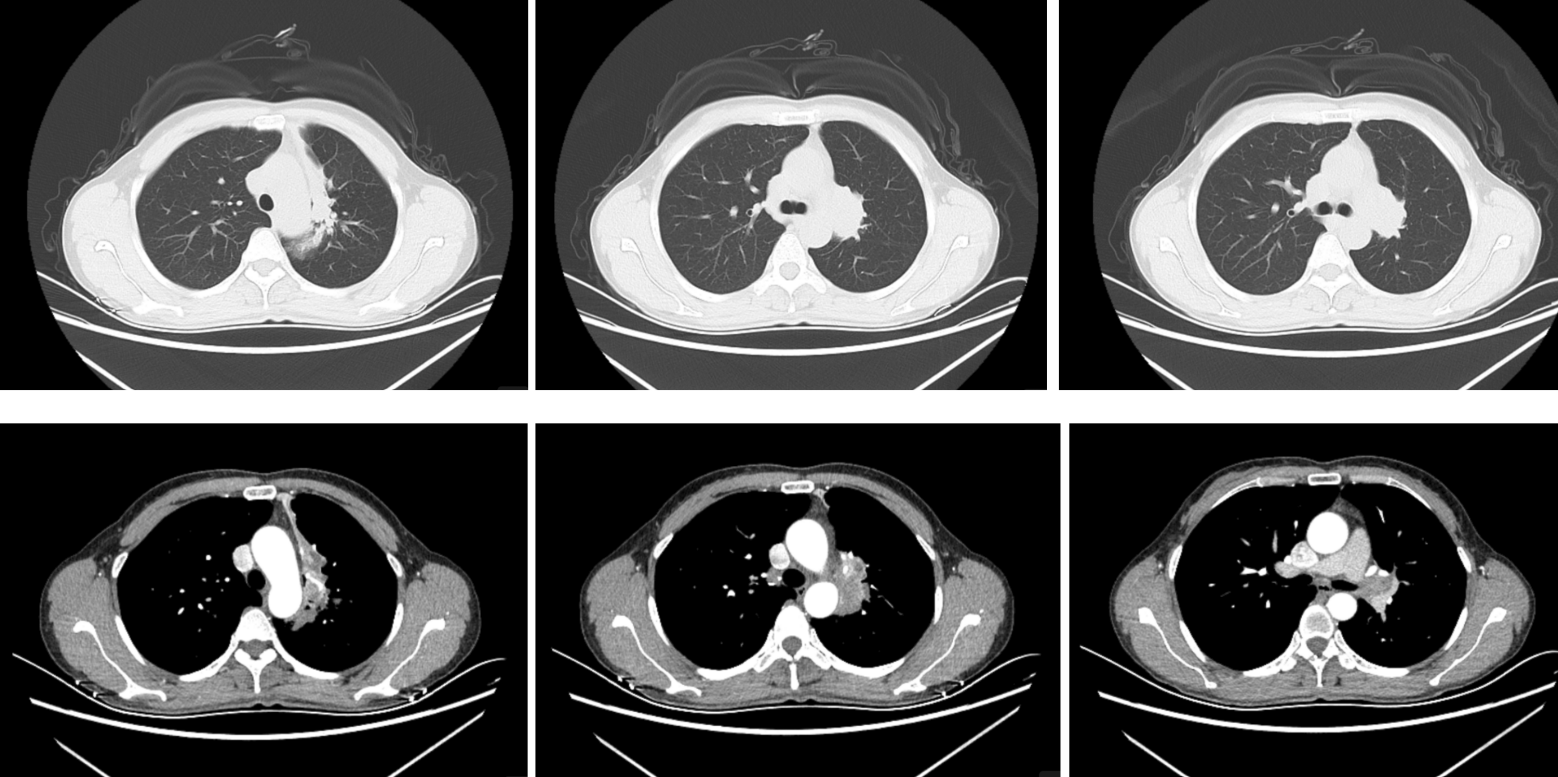
CT of chest showed a hyperenhancing mass in the left upper lobe.

**FIGURE 2:**
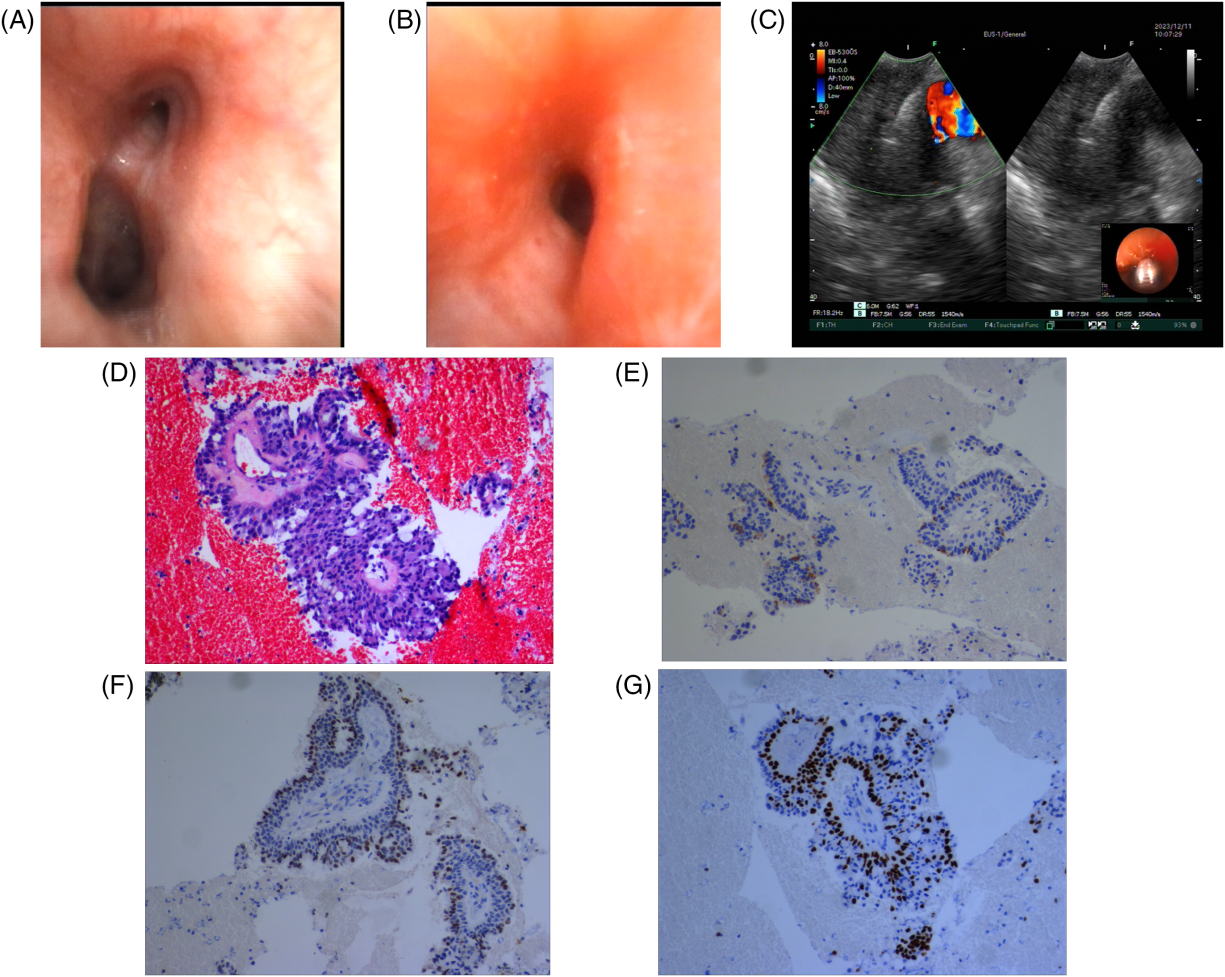
(A and B) Fibreoptic bronchoscopy showed that the left intrinsic segment bronchus was completely obstructed. (C) image of the mass under EBUS. (D) pathologic analysis revealed papillary structure with a fibrous vascular axis.(H‐E staining, ×100). (E) Napsin A positive in surface layer cells (×100). (F) TTF‐1 positive in surface layer cells (×100). (G) P40 positive in basal layer cells (×100).

A 57 year old woman presented with a 1‐month history of intermittent cough. Enhanced computed tomography (CT) of the chest showed a hyperenhancing mass in the left upper lobe (Figure 1). Fibreoptic bronchoscopy showed that the left main bronchus was completely obstructed (Figure 2A,B). However, the bronchial mucosa is smooth. Subsequently, endobronchial ultrasound‐transbronchial needle aspiration (EBUS‐TBNA) was performed (Figure 2C). The pathologic analysis revealed papillary structure with a fibrous vascular axis with H‐E (Haematoxylin‐Eosin) staining (Figure 2D). Immunohistochemical staining showed Napsin A (Figure 2E) and Thyriod transcription factor‐1/TTF‐1 (Figure 2F) positivity in surface layer cells and P40 (Figure 2G) positivity in basal cells.

## DISCUSSION

Solitary respiratory papilloma is a rare epithelial tumour that can be categorized into multiple subtypes. The glandular type (Glandular papilloma, GP) is the rarest,^1^, ^2^ which include lesions occurring in the bronchus and lung parenchyma. However, GP in lung parenchyma is extremely rare, with only a few cases previously reported.^3^, ^4^ The presenting symptoms are nonspecific, including cough or airway obstruction from large lesions. Treatment of GP differs, involving either interventional bronchoscopic therapy or surgical excision.

## AUTHOR CONTRIBUTIONS

ML Z and YH G were the lead author involved in drafting the initial manuscript and preparing the images. ML Z and CP Y provided radiological expertise including interpretation and description of the images. All authors contributed to the writing, review and final approval of the manuscript.

## CONFLICT OF INTEREST STATEMENT

None declared.

## ETHICS STATEMENT

Patient consent has been obtained prior to submission. Participant gave informed consent to participate in the study before taking part.

## Data Availability

Availability of data and materials The data that support the findings of this study are available on request from the corresponding author.
